# Hyperprogression: A novel response pattern under immunotherapy

**DOI:** 10.1002/ctm2.167

**Published:** 2020-09-23

**Authors:** Xue‐jiao Han, Aqu Alu, Yi‐nan Xiao, Yu‐quan Wei, Xia‐wei Wei

**Affiliations:** ^1^ Laboratory of Aging Research and Cancer Drug Target State Key Laboratory of Biotherapy National Clinical Research Center for Geriatrics West China Hospital Sichuan University Chengdu China; ^2^ West China School of Medicine West China Hospital Sichuan University Chengdu China

**Keywords:** checkpoint blockade therapy, HPD, hyperprogression, hyperprogressive disease, PD‐1/PD‐L1

## Abstract

Checkpoint blockade therapy has shown significant therapeutic benefits and resulted in durable responses in patients with various tumors. However, accumulating evidence has demonstrated that 4‐29% of all patients with cancers with various histologies may suffer from tumor flare following such therapy. This novel tumor response pattern, termed hyperprogression, is a potentially deleterious side effect of checkpoint blockade therapy that accelerates disease progression in a subset of patients. In this review, we describe possible immune checkpoint blockade biomarkers and the epidemiology, different definitions, and predictors of hyperprogression based on the research findings and further present the available evidence supporting pathophysiological hypotheses that might explain hyperprogression during checkpoint blockade therapy. We also compare hyperprogression and pseudoprogression. Finally, we discuss areas requiring further study.

## Graphical abstract

The occurrence of HPD, ranging from 4%∼29%, is not rare among cancer patients following ICB therapy.

The definition of HPD remains controversial.

The mechanism of HPD and reliable predictive markers require further exploration.

How to distinguish HPD from pseudoprogression is still a great challenge in clinical practice.

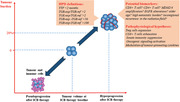

## 1 INTRODUCTION

Immune checkpoint blockade (ICB) therapy has profoundly revolutionized the treatment of various cancers, including melanoma,^1^ squamous and nonsquamous non‐small‐cell lung cancer (NSCLC),^2^ renal cell carcinoma,^3^ breast cancer,^4^ head and neck squamous cell carcinoma (HNSCC),^5^ urothelial carcinoma,^6^ and Hodgkin lymphomas.^7^ Immune checkpoint inhibitors (ICIs), especially monoclonal antibodies targeting programmed death‐1 (PD‐1) and cytotoxic T‐lymphocyte associated antigen‐4 (CTLA‐4), could reinvigorate exhausted T cells and have shown therapeutic benefits. The response rates achieved when using one anti‐PD‐1/PD‐L1 or anti‐CLTA‐4 antibody range from 10% to 30% for various cancers.^1^, ^2^, ^6^, ^8^ To date, the U.S. Food and Drug Administration has recommended the usage of six PD‐1/PD‐L1‐blocking monoclonal antibodies, including pembrolizumab, nivolumab, atezolizumab, durvalumab, avelumab, and cemiplimab, as well as one anti‐CLTA‐4 antibody, ipilimumab. In addition, TIM3, LAG3, and BTLA are inhibitory receptors of T cells, and their use needs further exploration.^9^

However, ICIs can induce novel tumor responses, such as pseudoprogression, which involves an initial increase in the size of tumor lesions with subsequent tumor shrinkage.^10^ The second atypical pattern of the response involves hyperprogression, which is characterized by accelerated disease progression and a reduced survival duration as a result of ICBs.^11^, ^12^, ^13^, ^14^ One case of a patient with stage IIB lung adenocarcinoma was the first report of hyperprogressive disease (HPD) based on the observation of tumor flare.^15^ Champiat et al first showed that the prevalence of HPD was 9% in patients following anti‐PD‐1/PD‐L1 therapy in phase I clinical trials, which aroused an interest in HPD.^16^ The determination of the tumor growth rate (TGR), tumor growth kinetics (TGK), and time to treatment failure (TTF) are useful algorithmic approaches to define hyperprogression. The rate of hyperprogression, depending on the different algorithmic methods used and the tumor type, ranges from 4% to 29%. However, the incidence, definition, predictors, and mechanisms of hyperprogression remain unknown to a large extent. The similarities between pseudoprogression and hyperprogression make it a challenge for clinicians to recognize hyperprogression to avoid the deleterious effects of therapy. Moreover, although there is a consensus that hyperprogression occurs mostly under the circumstances of ICB treatment, it sometimes also appears when treating patients with other therapeutic methods, such as chemotherapy and targeted therapy. As ICIs become more prevalent, it is urgent to improve the knowledge of this phenomenon to precisely determine the appropriate patients for immunotherapy. Therefore, we comprehensively reviewed ICB biomarkers and the epidemiology, definitions, and predictors of hyperprogression and compared them with those of pseudoprogression. We also clarified potential topics for further studies.

## 2 ICB THERAPY AND ITS PREDICTIVE BIOMARKERS

The emergence of ICBs, including anti‐CTLA‐4 antibodies and PD‐1/PD‐L1 checkpoint blockades approved by the U.S. Food and Drug Administration, has revolutionized the traditional treatment of many advanced malignancies and provided patients with more promising options due to the durable therapeutic effects, broad activity, and moderate toxicity profiles.^17^ Nevertheless, the limitations of ICBs, including a low response rate, immune‐related adverse events resulting from a hyperactive immune response, and primary and acquired resistance, have inevitably emerged and thus restrict its applicability in clinical practice.^18^ Consequently, the identification of predictive biomarkers for ICBs serves to distinguish patients who are likely to benefit from immunotherapy and to minimize adverse effects before treatment.^19^ Accumulating evidence has gradually shown the importance of taking the global features of the tumor into account when describing and assessing the ICB response and further indicates the four major factors and their intimate interactions: the tumor microenvironment; systemic immunity; tumor genome and epigenome; and environmental factors (gut microbiome).^20^

Due to the critical role of PD‐1/PD‐L1 in the final stage of antitumor response, most studies of predictive biomarkers have mainly focused on the tumor microenvironment and, in particular, have identified the expression levels of the ligands on tumor cells as potential biomarkers.^21^ While the predictive value of PD‐L1 in melanoma remains controversial, the U.S. Food and Drug Administration has updated the indications for PD‐1 inhibitors in the first‐line treatment of NSCLC, implying the importance of PD‐L1 as a biomarker (https://www.fda.gov/drugs/resources-information-approved-drugs/pembrolizumab-keytruda-checkpoint-inhibitor). However, assessment of PD‐L1 alone does not suffice to predict the clinical outcomes, since it may be affected by the use of different definitions of PD‐L1 positivity, the absence of a standard staining procedure to quantify expression levels, and the limited representativeness of samples resulting from sampling variability and tumor heterogeneity,^22^ all of which could partly account for the variance among the research results.

As a key component produced by activated T cells and Natural killer (NK) cells in the tumor microenvironment, interferon (IFN), including its downstream activated signaling pathways, plays an indispensable role in upregulating the expression level of PD‐L1/L2 on the tumor cell surface.^23^ The following studies have demonstrated the potential of members of the IFN family to be used for the assessment of the therapeutic effects of ICBs. One study analyzed the gene expression profiles of various tumor samples from patients treated with pembrolizumab, an anti‐PD‐1 antibody, and identified several immune‐related signatures, including IFN‐γ gene expression signatures, as predictive biomarkers of clinical benefits.^24^ Another study concerning the engineered humanized antibody MPDL3280A indicated a strong association between the elevated expression level of IFN‐c and downstream inducible genes in pretreatment tumors and the responsiveness to anti‐PD‐1/PD‐L1 therapy.^25^ However, durable IFN signaling could induce both PD‐L1‐dependent and PD‐L1‐independent resistance to ICBs by driving STAT1‐related epigenomic and transcriptomic changes in melanoma cells.^26^ In an open‐label, phase II clinical trial of NSCLC patients treated with atezolizumab, pre‐existing immunity, which was defined as IFN‐γ‐related gene expression, was correlated with improved overall survival.^27^ Given the contradictory results for the use of IFN‐γ as a predictive biomarker for ICBs, further studies regarding its modulation function in the complex antitumor response may serve to shed light on the applicability of IFN‐γ in clinical practice.

Neoantigens could be recognized as non‐self‐epitopes and reactivate the T cell‐mediated immune response against tumor cells as well as enhance the efficacy of ICBs.^28^ Therefore, neoantigens, together with somatic mutations in tumor cells, which are the direct cause of neoantigen formation, have been presumed to be possible predictive biomarkers for ICBs.^29^ In mouse models bearing progressive sarcomas, anti‐PD‐1 and anti‐CTLA‐4 agents could reactivate neoantigen‐specific T cells in tumors and induce tumor rejection.^30^ Enrichment of clonal neoantigens in lesions enhanced the sensitivity of patients with NSCLC or melanoma to anti‐PD‐1 and anti‐CTLA‐4 therapy and promoted overall survival in patients with lung adenocarcinomas.^31^ Similar positive correlations between tumor mutational burden (TMB) and significant clinical efficacy were observed among melanoma patients and NSCLC patients following ICB treatment.^32^, ^33^ However, the reliability of TMB as a predictive biomarker is restricted by its transcription process. Therefore, assessment of TMB or neoantigens alone could only provide limited reference value because many neoantigens and TMB are not closely associated with therapeutic benefits.^34^

In addition, further analysis regarding biomarkers on the surface of effector T cells as well as different T cell subsets in the peripheral blood also sheds light on their potential to reflect the response to ICBs. Among patients with advanced melanoma following ipilimumab treatment, an increase in the baseline number of CD4^+^CD25^+^FoxP3^+^ Tregs in the peripheral blood is correlated with significantly improved prognosis.^35^ Increases in the number of Ki‐67^+^ CD8 T cells and central memory CD4^+^CD27^+^CCR7^+^ T cells are potential biomarkers related to a positive clinical response to ICB therapies.^36^, ^37^ Because of the indispensable role of T cells in ICB therapies, only those neoantigens recognized by T cells, such as PPP1R3B and ATR in melanoma, could be factors predictive of therapeutic benefits^38^, ^39^; otherwise, neoantigens would be less effective as part of the antitumor response or even induce resistance to ICBs.^40^ As a prerequisite for the initiation of the T cell‐mediated antitumor response, HLA‐I molecules could also serve as candidate predictive biomarkers. When compared with homozygosity, heterozygosity at one HLA‐I locus could significantly enhance clinical outcomes in NSCLC or melanoma patients who received anti‐CTLA‐4 or anti‐PD‐1 therapies.^41^ The study further indicated that HLA‐B44 is related to extended overall survival, while the presence of HLA‐B62 or the absence of heterozygosity at HLA‐I might predict poor clinical outcomes in melanoma cohorts. T cell receptors responsible for recognizing the antigens presented by major histocompatibility complex (MHC) molecules have also attracted attention. In a pilot study of ipilimumab treatment, 12 melanoma patients with increased peripheral T cell receptor (TCR) diversity showed clinical benefits.^42^ Patients with a diverse subset of T cells and increases in tumor TCRs in the blood after treatment tended to show improved survival.^43^ However, current explorations of TCRs and their clinical implications are constrained by limited samples, and therefore, whether TCRs could be a reliable biomarker of ICB outcomes remains undetermined.

**FIGURE 1:**
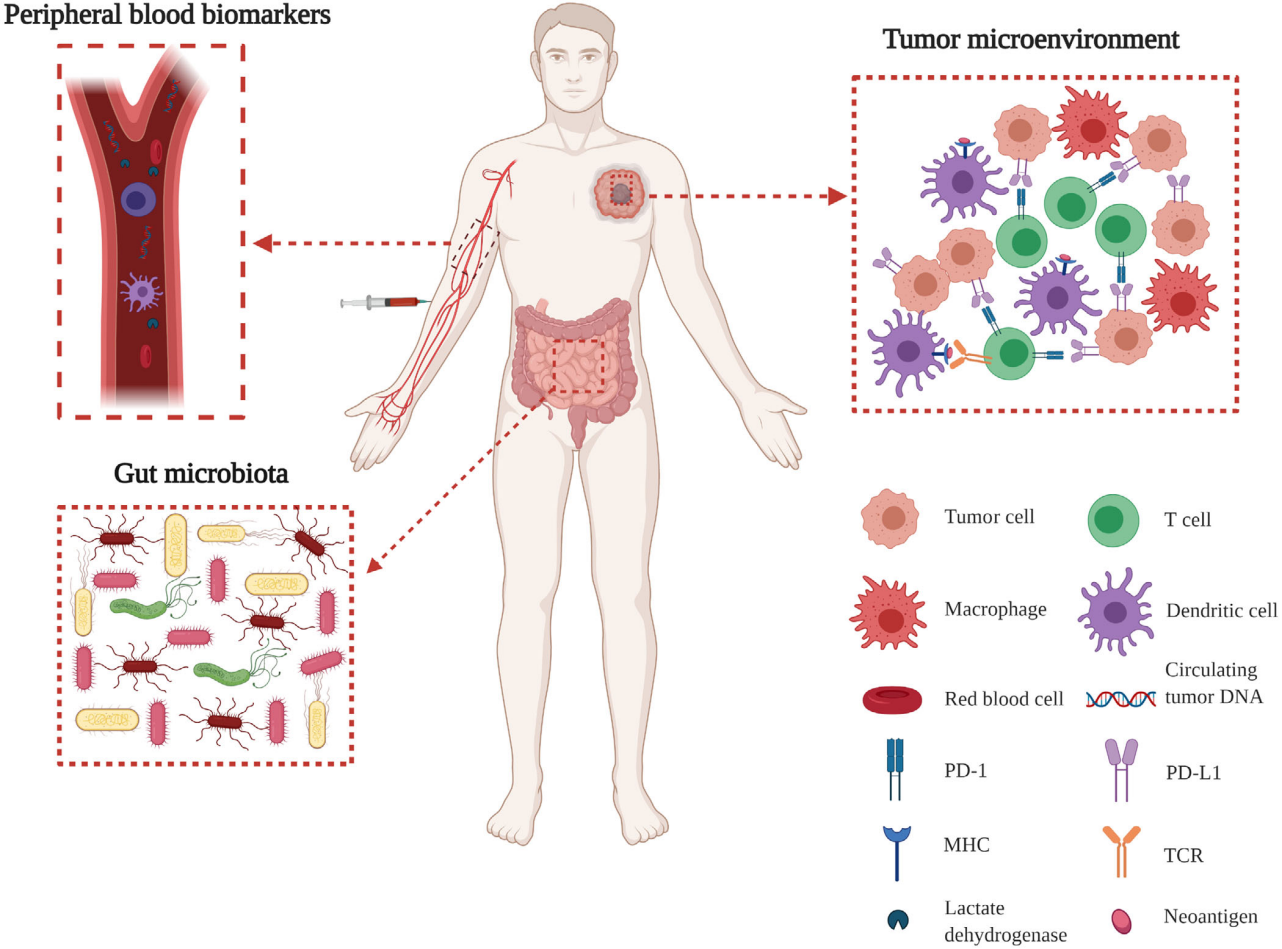
Illustration of potential biomarkers for checkpoint blockade therapy

Other widely investigated predictive biomarkers include tumor‐infiltrating immune cells,^44^ epigenetic modifications,^45^ peripheral blood biomarkers (lactate dehydrogenase,^46^ circulating tumor DNA,^47^ and immune‐cell counts^35^, ^48^), and microbiota^49^, ^50^ (Figure 1).

While the advent of ICB therapies and promising clinical trial results have offered patients with advanced tumors more hope and choices, many issues remain unresolved, including the assessment of indications, the low response rate among cancer patients, and potential toxicity. Therefore, biomarkers that could be used to predict and monitor response patterns to certain immunotherapies will facilitate personalized treatment in the near future to maximize the therapeutic benefits as well as minimize the occurrence of immune‐related adverse events. Furthermore, insights from these seminal works regarding the sophisticated mechanisms underlying antitumor responses might identify additional combination therapy strategies and clarify the appropriate management of ICBs.

## 3 ANECDOTAL PHENOMENON ASSOCIATED WITH CHECKPOINT BLOCKADE THERAPY

Over the past few years, ICBs have begun to transform clinical cancer management owing to their capability for remarkably increasing 10‐year survival and decreasing adverse effects.^51^ Since anti‐PD‐1/PD‐L1 monotherapy has resulted in durable tumor responses and improved clinical outcomes when used alone or in combination with other therapies,^2^, ^52^ these agents are more widely applicable in clinical practice. Nevertheless, previous studies failed to identify fully reliable biomarkers, including PD‐L1 expression^53^, ^54^ and microsatellite instability,^55^ that could distinguish patients who are likely to respond to and benefit from ICBs. As an increasing number of cancer patients are receiving ICB therapy, an increasing number of hyperprogression cases have been observed as well (Table 1). As first reported by Chubachi and Yasuda in 2016, hyperprogression was observed during anti‐PD‐1 monotherapy of lung adenocarcinoma.^15^ Seven years after tumor recurrence, the patient was administered nivolumab (3 mg/kg, every 2 weeks) as the 10th line of therapy. The disease was indolent before nivolumab treatment. However, 6 weeks later, the patient showed multiple novel nodules in the lungs and brain upon receiving whole‐body CT and MRI of the head to assess treatment response. In addition, Xu et al presented a unique case of a patient with cervical small cell carcinoma who developed hyperprogression after treatment with pembrolizumab and showed a correlation between the AKT1 E17K mutation and HPD.^56^ The 49‐year‐old woman received pembrolizumab (150 mg, every 3 weeks) after surgery and chemotherapy. However, MRI imaging revealed a greater than 50% increase in the volume of pelvic lesions and new metastases 2 months after pembrolizumab treatment. The researchers sequenced the whole exome of patients and found a high prevalence of the AKT1 E17K mutation (26%) in tumor tissue. Moreover, dynamic monitoring of circulating tumor DNA (ctDNA) showed that the mutation rate of AKT1 E17K in peripheral blood increased successively and was correlated with tumor growth, suggesting that the mutation was a possible molecular mechanism underlying HPD in cervical small cell carcinoma.

**TABLE 1:** A summary of the published case reports on hyperprogression after checkpoint blockade therapy

Year	Gender	Age (years)	Tumor type	Tumor stage	Agents	References
2016	Male	54	Lung adenocarcinoma	IIB	Nivolumab	^15^
2017	Female	61	Squamous NSCLC	IIIB	Nivolumab	^188^
2017	Male	67	Squamous NSCLC	IIIA	Nivolumab	^188^
2018	Male	76	Lung adenocarcinoma	IVB	Pembrolizu‐mab	^189^
2018	Male	77	Anorectal melanoma	IIB	Pembrolizu‐mab	^190^
2018	Female	13	Melanoma	IIID	Nivolumab	^191^
2018	Female	60	NSCLC	IV	Nivolumab	^192^
2018	Male	69	Squamous NSCLC	IIIB	Nivolumab	^193^
2018	Male	83	Lung PC	IIIA	Nivolumab	^193^
2018	Female	74	Lung PC	IVA	Nivolumab	^193^
2018	Female	53	Lung adenocarcinoma	IVB	Nivolumab	^193^
2018	Male	80	Squamous NSCLC	IVB	Nivolumab	^193^
2018	Male	66	Gastroesophageal cancer	IV	Nivolumab	^194^
2018	‐	‐	ATLL	‐	Nivolumab	^132^
2019	Male	71	HCC	‐	Nivolumab	^195^
2019	Male	71	HCC	‐	Nivolumab	^195^
2019	Male	57	HCC	‐	Nivolumab	^195^
2019	Male	69	HCC	‐	Tremelimu‐mab	^195^
2019	Male	72	HCC	‐	Nivolumab	^195^
2019	Male	69	HCC	‐	Tremelimu‐mab and Durvalumab	^195^
2019	Male	86	UBC	T2G3	Pembrolizu‐mab	^196^
2019	Female	65	UBC	T2G3	Pembrolizu‐mab	^196^
2019	Male	58	UBC	TaG3	Anti‐PD‐L1	^197^
2019	Female	60	HNSCC	IVB	Nivolumab	^198^
2020	Male	37	HCC	‐	Pembrolizu‐mab	^199^

Abbreviations: ATLL, adult T‐cell leukemia‐lymphoma; HCC, hepatocellular carcinoma; HNSCC, head and neck squamous cell carcinoma; NSCLC, non‐small‐cell lung cancer; PC, pleomorphic carcinoma; UBC, urothelial bladder carcinoma.

Later, some retrospective studies demonstrated that hyperprogression was not a rare side effect among patients receiving ICB treatment (Table 2). For example, in a clinical study involving 218 patients following anti‐PD‐1/PD‐L1 therapies, it was discovered that 9% of these patients presented HPD.^16^ HPD was defined as a ≥ two‐fold increase in TGR between the anti‐PD‐1/PD‐L1 treatment and reference periods (before treatment onset) upon comparison of CT scans. However, because of some limitations of the diagnostic criteria of HPD, 18 of the 218 patients who showed disease progression and a high TGR in new lesions were found not to have HPD. Therefore, the authors suggested that the frequency of HPD might be higher than the reported 9% rate.^16^ Moreover, Ferrara and his colleagues focused on the occurrence of HPD in advanced NSCLC by comparing patients receiving PD‐1/PD‐L1 inhibitors with those treated with single‐agent chemotherapy. HPD was defined as a change in TGR expression greater than 50% each month. The rate of HPD in patients receiving PD‐1/PD‐L1 inhibitors was 13.6%, compared to 5.1% for patients treated with chemotherapy. In addition, it was reported that HPD was closely related to high metastatic burden but not the baseline tumor burden.^57^ Furthermore, Kim et al defined HPD as a ≥ two‐fold increase in TGR, TGK, or TTF over less than 2 months. They further indicated that the occurrence of HPD was 20.9%, 20.5%, and 37.3% when assessed according to TGK, TGR, and TTF, respectively.^58^

**TABLE 2:** Main retrospective studies on hyperprogression receiving immune checkpoint inhibitors

Participants	Cancer type	Agents	HPD defined	Rates	References
Single institution	All	PD1/PD‐L1 inhibitors	TGR > 2 according to tumor volume	9.1% (12/ 131)	^11^
Single institution	All	CTLA‐4 and PD‐1/PD‐L1 inhibitors, other investigational agents	TTF < 2 months; Tumor burden > 50%; Progression pace > 2x	3.8% (6/ 155)	^61^
Multicenter	HNSCC	PD1/PD‐L1 inhibitors	TGK > 2 according to RECIST1.1	29.4% (10/ 34)	^72^
Multicenter	NSCLC	PD1/PD‐L1 inhibitors	Variation of TGR > 1.5 according to tumor volume	13.8% (56/ 406)	^57^
Single institution	All	CTLA‐4 and PD‐1/PD‐L1 inhibitors	‐	11.0% (6/ 56)	^178^
Single institution	All	PD1/PD‐L1 inhibitors and other checkpoint inhibitors	TGR > 2 according to tumor volume	7.0% (12/ 182)	^75^
Single institution	NSCLC	PD1/PD‐L1 inhibitors	TGK > 2 or TGR > 2 or TTF < 2 months	20.9% (55/ 263) 20.5% (54/ 263) 37.3% (98/ 263)	^58^
Single institution	AGC	PD1/PD‐L1 inhibitors	TGR > 2	21.0% (13/ 62)	^73^

Abbreviations: AGC, advanced gastric cancer; HNSCC, head and neck squamous cell carcinoma; HPD, hyperprogressive disease; NSCLC, non‐small cell lung cancer; TGK, tumor growth kinetics; TGR, tumor growth rate; TTF, time to treatment failure.

## 4 DIFFERENT HPD DEFINITIONS

There are five different definitions used to assess HPD during ICB therapy. Champiat et al defined HPD as progression at the first evaluation according to Response Evaluation Criteria In Solid Tumors (RECIST) and an at least a two‐fold increase in the TGR between the reference (REF) and the experiment (EXP) periods.^16^ Briefly, TGR refers to the percentage of variation in tumor volume over 1 month. Similarly, a study by Saâda‐Bouzid et al defined HPD as a TGK_R_ ≥ 2 based on a linear tumor growth model and expressed it as a ratio to minimize the overestimation of the rate of HPD, which could occur because of a larger tumor volume generated by the doubling of size in one dimension.^59^ Singavi and colleagues adopted the same definition used by Champiat et al and included the criterion of an additional 50% increase in tumor size according to RECIST during ICI treatment.^60^ Ferrara et al defined HPD as a ΔTGR (the difference between TGR during and before treatment) exceeding 50%.^57^ Finally, the study by Kato et al defined HPD according to three criteria: a > two‐fold increase in progression pace, a TTF < 2 months, and a > 50% increase in tumor burden according to RECIST during ICB therapy.^61^

To date, no consensus HPD definition has been made, leading to a risk of inconsistency in the description of different tumor behaviors. Therefore, Kas and colleagues conducted a retrospective cohort study including 406 patients with advanced NSCLC treated with PD‐1/PD‐L1 inhibitors to assess the accuracy of each definition and identify the incidence of HPD as well as the association between each HPD definition and overall survival.^62^ They found that according to five different definitions, HPD incidence varied from 5.4% to 18.5% in the cohort. ΔTGR was closely correlated with poor overall survival. Therefore, ΔTGR might be the most accurate parameter when used to distinguish HPD patients from non‐HPD progressors. Considering that the concept of HPD involves both a great increase in tumor kinetics and a poor survival outcome, the researchers proposed a new definition of HPD: a RECIST percentage during therapy greater than 20% and a ΔTGR greater than 100%. However, there are some limitations. For example, measurable lesions are defined according to the RECIST1.1 criteria in this study, which do not account for the appearance of new lesions and the unequivocal progression of nontarget lesions. The tumor response toward immunotherapies is different from that toward cytotoxic drugs or targeted agents due to the observed novel patterns of response and disease progression.^63^ For instance, a subgroup of patients meeting the RECIST1.1 criteria for disease progression^64^ have been proven to show delayed but durable responses.^65^, ^66^ Researchers have developed several immune‐related response criteria, such as the immune‐related response criteria,^67^ immune‐related RECIST,^68^ and immune RECIST.^69^ The differences between them are summarized in Table 3. Therefore, the discrimination of HPD remains a major challenge and needs further study.

**TABLE 3:** Comparison of irRC, irRECIST, and iRECIST

	irRC	irRECIST	iRECIST
Measurement Modality	Bidimensional (Longest Diameter × Longest Perpendicular Diameter)	Unidimensional (Longest Diameter)	Unidimensional (Longest Diameter)
Measurable lesions	5 mm × 5 mm	≥10mm	≥10mm
Numbers and site of target disease	10 lesions in total; 5 per organ	5 lesions in total; 2 per organ	5 lesions in total; 2 per organ
Appearance of new lesions	Incorporated into tumor burden	Incorporated into tumor burden	iUPD becomes iCPD if PD is eventually confirmed
CR	Disappearance of all lesions	Disappearance of all lesions	Disappearance of all lesions
PR	≥50% decrease in tumor burden compared with baseline	≥30% decrease in tumor burden compared with baseline	≥30% decrease in tumor burden compared with baseline
SD	Neither CR nor PD is met	Neither CR nor PD is met	Neither CR nor PD is met
PD	≥25% increase in the nadir of the sum of target lesions	≥20% increase in the nadir of the sum of target lesions (with a minimum of 5 mm)	≥20% increase in the nadir of the sum of target lesions (with a minimum of 5 mm)
Confirmation of PD	Yes, at least 4 weeks later	Yes, at least 4 weeks after and up to 12 weeks	Yes, at least 4 weeks after and up to 8 weeks
References	^67^	^68^	^69^

Abbreviations: CR, complete response; iCPD, immune confirmed progressive disease; irRC, immune‐related response criteria; irRECIST, immune‐related RECIST; iRECIST, immunotherapy RECIST; iUPD, immune unconfirmed progressive disease; PD, progressive disease; PR, partial response; SD, stable disease.

## 5 HPD OCCURS IN VARIOUS TUMORS

HPD occurs in various tumors. The most commonly studied cancer type is NSCLC. Early evidence has been obtained from phase III clinical trials (Checkmate 057 and Checkmate 026 trials).^70^, ^71^ In addition, several retrospective studies also focused on the occurrence of HPD in advanced NSCLC patients.^57^, ^58^ Ferrara and his colleagues reported that the rate of HPD for NSCLC patients treated with PD‐1/PD‐L1 inhibitors was 13.6%.^57^ The incidence of HPD among HNSCC patients is 29%.^72^ Furthermore, among 62 patients with advanced gastric cancer treated by nivolumab, 13 developed HPD based on the definition of HPD as consisting of a two‐fold increase in the TGR.^73^ However, in another study, the occurrence of HPD in advanced gastric cancer patients after nivolumab treatment was ∼10%.^74^ In phase III clinical trials, patients with urothelial carcinoma developed HPD after anti‐PD‐1/PD‐L1 treatment.^6^, ^8^ In summary, HPD might actually occur in all types of tumors despite the lack of evidence in certain types of cancer.^11^, ^61^, ^75^

## 6 POTENTIAL PREDICTORS OF HPD

**FIGURE 2:**
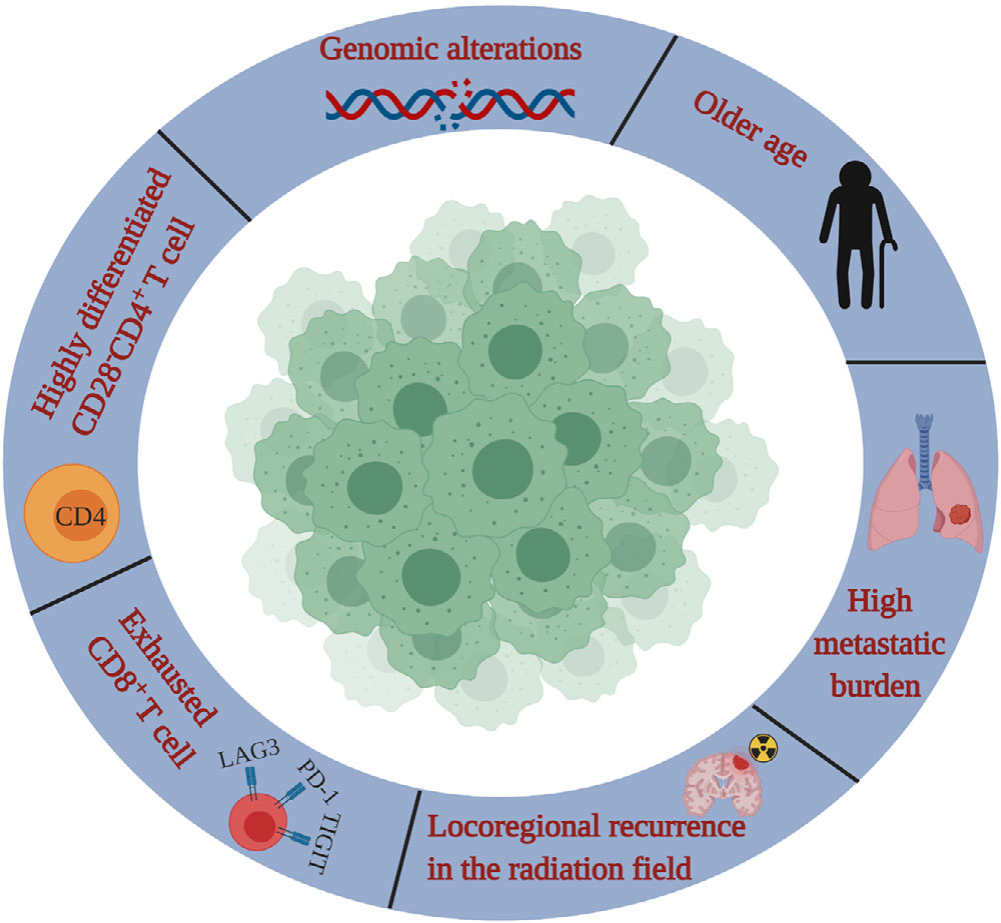
Illustration of possible factors to predict hyperprogression under immunotherapy

Since immunotherapy is quite common in patients with advanced tumors, it is necessary to identify the biomarkers of HPD to select patients carefully and avoid the deleterious effects of therapy. A few factors might be potential predictors of HPD, such as the phenotypes of CD8^+^ and CD4^+^ T cells, MDM2/MDM4 amplification, EGFR alterations,^58^, ^61^ and other factors, including older age,^16^ high metastatic burden,^57^ and locoregional recurrences in the radiation field (Figure 2).^13^ However, the tumor burden at baseline as well as PD‐L1 expression in tumors is an irrelevant factor in HPD.^15^, ^16^, ^57^

Kim and his colleagues focused on CD8^+^ T lymphocytes in the peripheral blood to seek potential predictors.^58^ Intriguingly, they found that the number of effector/memory CD8^+^ T lymphocytes (CCR7^−^CD45RA^−^)^76^ decreased, while that of exhausted tumor‐reactive CD8^+^ T lymphocytes (TIGIT^+^ PD‐1^+^)^77^ increased in HPD patients with NSCLC. Additionally, these two biomarkers independently predicted clinical outcomes based on progression‐free survival and overall survival. The results indicate that the degree of pre‐existing antitumor immunity and the severity of T cell exhaustion can be predictors for HPD. The exhaustion of CD8^+^ T cells in the tumor immune microenvironment is one of the potential mechanisms that triggers the acceleration of tumor growth under ICB treatment. The blockade of PD‐1 may lead to the overexpression of other immune checkpoints, such as TIGIT and LAG3.^78^

In addition to CD8^+^ T cells, CD4^+^ T cells are now considered potential predictors of HPD. Arasanz et al^79^ recently confirmed that systemic expansion of highly differentiated CD28^−^CD4^+^ T lymphocytes (CD4^+^T_HD_) was a potential biomarker of HPD and associated with poor clinical outcomes in NSCLC patients receiving immunotherapy. The expression of CD28, which is often present on naive T cells, is a marker of differentiation.^80^ HPD patients showed a significant elevation in the number of CD4^+^ T_HD_ cells that was above the baseline standard after a cycle of immunotherapy. The change in the CD4^+^ T_HD_ cell proportion of 1.3 between posttreatment and pretreatment could correctly distinguish HPD patients with 82% specificity and 70% sensitivity. Therefore, real‐time monitoring of CD4^+^ T_HD_ cells allows early detection of HPD in combination with radiological examination, which might improve the prognosis in clinical practice.^79^ The effectiveness of the response of NSCLC patients to anti‐PD‐1/PD‐L1 immunotherapy requires functional systemic CD4 immunity at the baseline level, which could enhance the proliferative capacities of CD8^+^ T cells.^81^ Patients who are responsive to ipilimumab and GM‐CSF experienced a significant elevation in specific clusters of CD4^+^ T cells expressing decreased levels of CD127 and PD‐1, which are markers suggestive of the functional activity of T cells.^82^ Moreover, the number of CD62L^low^CD4^+^ effector‐memory Th1 cells is also significantly higher in the peripheral blood of responders with NSCLC before PD‐1 blockade, whereas a decrease in CD62L^low^CD4^+^ T cells is associated with acquired resistance.^83^ All these results present new opportunities to evaluate the use of CD4^+^ T cell immunity as a powerful predictor, even more powerful than the use of CD8^+^ T cell immunity, of responders with multiple tumor types receiving immunotherapy.

Several studies have demonstrated that ICBs are not as effective in NSCLC patients harboring EGFR mutations or ALK rearrangements.^84^ Therefore, researchers are attempting to determine whether these genomic alterations could be risk factors for HPD. Since Chinese patients with lung adenocarcinoma have a much higher rate of EGFR mutations than Caucasians,^85^ researchers evaluated Chinese patients treated with single‐agent ICIs and found that EGFR/ALK alterations were associated with HPD.^86^ This finding is consistent with another study of NSCLC patients treated with anti‐PD‐1/PD‐L1 therapy.^87^ In fact, EGFR activation contributes to the upregulation of PD‐1, PD‐L1, and CTLA‐4 expression levels as well as the decrease in T cell infiltration and reduces inflammation in the tumor microenvironment.^87^ On the other hand, researchers investigated potential genomic markers associated with HPD during ICB treatment by next‐generation sequencing. Importantly, they found that patients with MDM2/MDM4 amplification or EGFR aberrations showed an increased TGR after receiving ICB treatment.^61^ MDM2 amplification has been reported in multiple tumor types.^88^ This group also analyzed MDM2 amplification in 102 878 patients with different malignancies by next‐generation sequencing and found that 3.5% of patients had MDM2 amplification.^89^ Regarding the mechanism, the authors hypothesized that ICBs might activate JAK‐STAT^90^ through IFN‐γ,^91^ subsequently increasing the expression of IRF‐8,^92^ which could induce MDM2 expression.^93^ The core functional domain of MDM2 could recognize the N‐terminal transactivation domain and subsequently inhibit the tumor suppressor at the transcriptional level. MDM2 amplification promotes proteasomal degradation of p53 and contributes to tumorigenesis.^94^ However, in another study on patients with advanced gastric cancer, non‐HPD patients also possessed genetic changes, such as MDM2 amplification, ERBB2 amplification, KRAS amplification, TP53 mutations, and PIK3CA mutations, implying that these changes might not be specific biomarkers of HPD.^74^

In fact, genomic alterations are highly correlated with the immunotherapeutic response. For example, a high TMB level is a biomarker useful for selecting suitable patients for ICB therapy. This is because mutations may generate immunogenic neoantigens, which could facilitate the recognition of cancer cells as foreign invaders. Accumulating evidence has suggested that patients with tumors harboring a higher mutational burden are more prone to show survival benefits after ICI treatment.^95^, ^96^ However, in a case of cervical small cell carcinoma,^56^ a patient who was assessed as eligible and suitable for ICB therapy before initiating pembrolizumab treatment still suffered from tumor hyperprogression. The whole exome sequence and ctDNA mutation analysis indicated that the rate of AKT1 E17K mutation accumulation successively increased, which was consistent with tumor growth. Is it possible that mutations generated from ICB therapy led to HPD? Cytotoxic chemotherapy can also produce subclonal neoantigens, but patients have reduced clinical benefits from therapy.^31^ Therefore, further studies are needed to clarify the relationship between genomic alterations and HPD.

Besides, some other factors might be associated with HPD, including older age, high metastatic burden, and locoregional recurrence in the radiation field. The different immunological backgrounds, such as a general reduction in T cell immunity,^97^ or increases in myeloid‐derived suppressor cells or Treg prevalence might account for the vulnerability of the elderly to HPD.^98^, ^99^ However, this association varies in different studies.^14^, ^57^, ^58^ Ferrara et al^57^ found that HPD was correlated with the presence of two or more metastatic sites before anti‐PD‐1/PD‐L1 therapy in comparison with non‐HPD (62.5% vs 42.6%). It seems reasonable to postulate that the more aggressive a tumor is, the higher the risk for HPD will be. However, there is no evidence regarding the association with the baseline tumor burden found in different studies. Interestingly, patients with HPD showed a slower progression pace and a lower rate of new lesions before immunotherapy initiation in another study.^16^ Since the study by Ferrara and colleagues is the only work presenting such evidence, more studies are required. The association between HPD and locoregional recurrence in the radiation field has been documented only in patients with HNSCC and has failed to be observed in other studies.^72^

## 7 PATHOPHYSIOLOGICAL HYPOTHESES OF HPD

**FIGURE 3:**
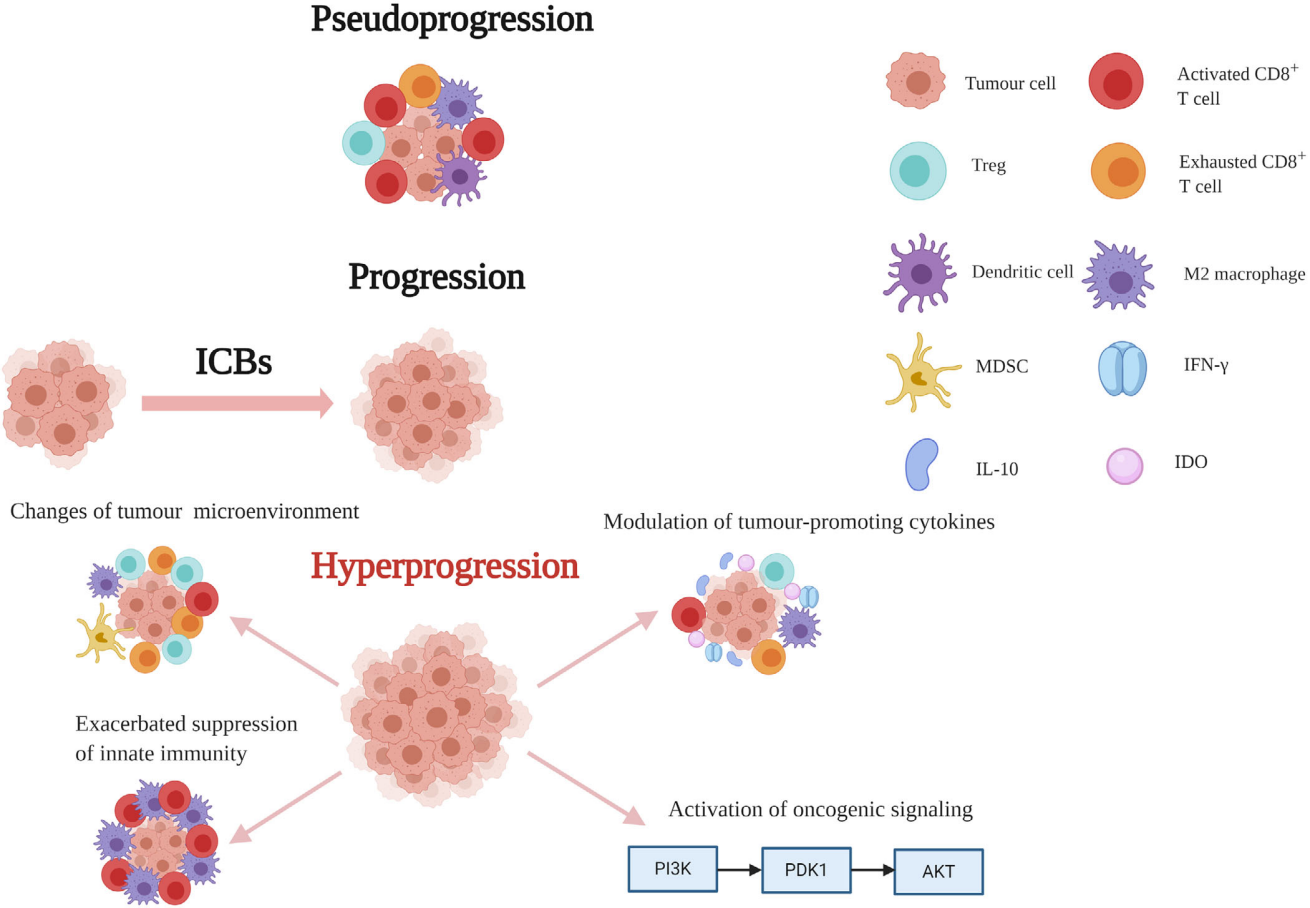
Schematic diagram illustrates pseudoprogression and hyperprogression under immune checkpoint blockade therapy, along with the pathophysiological hypotheses for hyperprogression

The pathophysiological mechanisms of HPD remain largely unknown. However, an increasing number of studies has demonstrated that changes in the tumor immune microenvironment during checkpoint blockade therapy, such as activation of PD‐1‐expressing Treg cells and CD8^+^ T cell exhaustion, could trigger the acceleration of tumor growth. Moreover, the exacerbation of the suppression of innate immunity, activation of oncogenic signaling, and modulation of tumor‐promoting cytokines may be crucial to the occurrence of HPD (Figure 3).

### 7.1 Changes in the tumor immune microenvironment

Indeed, immune checkpoints are expressed both on the surface of tumor‐active CD8^+^ T cells and tumor‐specific Treg cells in the tumor microenvironment.^100^, ^101^ Therefore, PD‐1 blockade immunotherapy could activate and foster the growth of tumor‐specific Treg cells and tumor‐activated CD8^+^ T cells.^102^, ^103^ Kamada and his colleagues recently found that in HPD patients with advanced gastric cancer, tumor‐infiltrating PD‐1^+^ FoxP3^high^CD45RA^−^CD4^+^ T (eTreg) cells were activated after anti‐PD‐1 treatment.^74^ In addition, they proved that anti‐PD‐1 therapy augmented the proliferation and inhibited the suppressive activity of Treg cells in vitro and in mice. Similarly, in malignant melanoma patients receiving anti‐PD‐1 therapy, the number of Treg cells was increased in nonresponders, in contrast with the reduction in responders.^104^ Moreover, combination therapy with anti‐PD‐1 and anti‐CTLA‐4 monoclonal antibodies was reported to deplete Treg cells^105^, ^106^ and decrease the incidence of HPD in melanoma patients.^107^ On the other hand, the evidence also revealed that Treg cells could play essential roles in other diseases, such as chronic viral infection. For example, an anti‐PD‐1 agent contributed to the expansion and function of Treg cells in the livers of patients with chronic HCV infection.^108^ In addition, HIV infection could increase the expression of PD‐1 in Treg cells, and PD‐L1 blockade could restore the proliferation of Treg cells in viremic individuals.^109^ In conclusion, there is a possibility that PD‐1 blockade immunotherapy leads to a substantial increase in Treg cells and ultimately results in accelerated tumor growth in HPD patients. Therefore, patients receiving anti‐PD‐1 therapy could benefit from the monitoring of changes in Treg cells in clinical practice.

**FIGURE 4:**
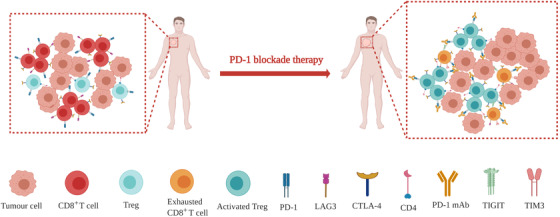
Schematic diagram illustrates changes of tumor immune microenvironment after PD‐1 blockade therapy, including Treg cells expansion and CD8^+^ T cells exhaustion

PD‐1 is not necessary for the induction of CD8^+^ T cell exhaustion.^78^, ^110^ More importantly, in mice with chronic virus infection, the absence of PD‐1 led to the overexpression of other immune checkpoints, such as LAG3 and TIGIT. In addition, the absence of PD‐1 might also contribute to excessive proliferation and differentiation of exhausted CD8^+^ T cells in the final stage.^78^ Similarly, in an ovarian cancer mouse model, PD‐1 blockade conferred compensatory enhanced expression of LAG3 and CTLA‐4 on CD8^+^ T cells.^111^ Furthermore, Koyama et al analyzed the tumor immune microenvironment in a lung adenocarcinoma mouse model and two NSCLC patients following anti‐PD‐1 treatment.^112^ They observed the upregulation of TIM3 in tumor‐infiltrating cytotoxic CD8^+^ T cells after treatment failure. Moreover, combined immunotherapy is more effective in controlling tumor growth than anti‐PD‐1 monotherapy.^113^, ^114^ These results indicate that compensatory immune suppression and escape activated by anti‐PD‐1/PD‐L1 therapy may result in hyperprogression. Kim et al directly showed that the number of severely exhausted tumor‐reactive CD8^+^ T cells was increased in patients with HPD. However, the mechanisms of the interaction between various immune checkpoints remain largely unknown. As they are characterized by a loss of effector functions and proliferation as well as an altered transcriptional programme, exhausted T cells usually accumulate following prolonged antigen stimulation.^115^, ^116^ The upregulation of immune checkpoints, including PD‐1, TIM3, LAG3, CTLA‐4, and TIGIT, is one of the hallmarks of T cell exhaustion.^115^, ^117^, ^118^ Exhausted T cells display functional impairment in their production of effector cytokines in multiple cancers,^115^, ^119^, ^120^, ^121^ which restricts the immune response to cancer cells (Figure 4).

### 7.2 Exacerbated suppression of innate immunity

It is now clear that PD‐1 blockade has a negative interaction with the innate immune system. On the one hand, myeloid‐originated immune cells with high PD‐L1 expression in the tumor microenvironment alleviated the efficacy of ICIs by competitive binding of anti‐PD‐1 antibodies with T lymphocytes or the secretion of immunosuppressive molecules.^122^ For example, under the circumstances of PD‐1 inhibition, we observed the decreased generation of lytic molecules, such as perforins and granzymes, in NK cells^123^ as well as increased immunosuppressive cytokine IL10 secretion from tumor‐infiltrating dendritic cells and monocytes.^124^, ^125^ Additionally, anti‐PD‐L1 administration may trigger the accumulation of immunosuppressive M2 macrophages in tumor sites characterized by the “M2” markers CD163 and PD‐L1 in different cancers, including NCSLC, colorectal cancer, breast cancer, and cervical cancer,^126^, ^127^, ^128^, ^129^ and this macrophage accumulation may worsen the prognosis during ICB treatment. In a recent study performed by Lo Russo et al,^130^ the infiltration of M2‐like CD163^+^CD33^+^PD‐L1^+^ clustered epithelioid macrophages was observed in tissue samples from 39 NSCLC patients with HPD after PD‐1/PD‐L1 blockade as well as in tumor lesions from immunodeficient mice inoculated with patient‐derived xenografts and human lung cancer cells. The induction of HPD by tumor‐associated macrophage reprogramming is reliant on the antibody‐Fc/FcR interaction on macrophages rather than anti‐PD‐1 F(ab)2 fragments in the environment of ICIs. Thus, this distinctive immunophenotype may be a potential predictor of HPD.

### 7.3 Activation of oncogenic signaling

Wartewig et al reported that anti‐PD‐1/PD‐L1 therapy could induce HPD in mouse models with T‐cell non‐Hodgkin's lymphoma.^131^ They demonstrated that PD‐1 could suppress oncogenic signaling by upregulating PTEN levels while attenuating PI3K/AKT and NF‐κB signaling. Thus, PD‐1 inhibitors could accelerate T cell growth rapidly. In concordance with this preclinical study, hyperprogression was also observed in three patients with the chronic, smouldering, and acute subtypes of lymphoma and adult T cell leukemia after a single dose of nivolumab.^132^ Only one study reported the PD‐1 expression level and its positive effects on the progression of melanoma,^133^ and more research is needed to confirm this issue. Oncogenic RAS signaling not only increases PD‐L1 mRNA stability^134^ but also leads to mutations of KRAS and TP53 in lung adenocarcinoma, which may imply a reaction to anti‐PD‐1 therapy.^135^ Moreover, MYC‐induced oncogenic stress could initiate immune escape by regulating CD47 or PD‐L1.^136^ Constitutive activation of ERK might downregulate tristetraprolin, a crucial protein responsible for the stability of IL8/CXCL8 mRNA in melanoma.^137^ On the other hand, amplification of MDM2 and EGFR mutations is related to hyperprogression among patients with advanced solid cancers during ICB treatment,^12^ and PD‐1 blockade could trigger immune escape in lung cancers driven by EGFR, as shown in preclinical studies.^138^ Taken together, the evidence suggests that it is highly likely that PD‐1/PD‐L1 inhibition plays a critical role in oncogenic signaling pathways.

### 7.4 Modulation of tumor‐promoting cytokines

As found in preclinical studies, PD‐1 blockade immunotherapy could stimulate tumor‐infiltrating DCs to secrete IL‐10, which subsequently upregulated PD‐1 in a STAT‐3‐dependent manner on DCs, hence creating a vicious circle of immune escape.^124^ In addition, PD‐1 inhibition augmented the expression of the IL‐10 receptor by regulating tumor‐specific CD8^+^ T cells in the peripheral blood of patients diagnosed with advanced melanoma.^139^ IL‐10 impeded antigen presentation and costimulation, which inhibited antigen‐specific T cell responses.^140^, ^141^ On the other hand, PD‐1 blockade also increased the serum concentration of angiopoietin 2 in advanced melanoma, which was related to M2 macrophage infiltration.^142^ Finally, IFN‐γ secretion also increased as a result of the inhibition of PD‐1/PD‐L1 in mouse models,^91^ and extensive CD4^+^ T cell infiltration resulting from PD‐1 silencing contributed to the increased production of IFN‐γ in mouse models of *Mycobacterium tuberculosis* compared with that in wild‐type mice.^143^ Although a large amount of data suggest that IFN‐γ acts as a key factor in anticancer immunity,^144^, ^145^ there is significant evidence indicating that IFN‐γ promotes immune escape.^146^ IFN‐γ has been reported to stimulate MDSC development^147^ and induce indoleamine 2,3‐dioxygenase expression, which leads to the induction of Treg cells.^148^, ^149^ Therefore, constant activation of IFN‐γ signaling might mitigate both cancer immunoediting and tolerance to ICBs.^26^

## 8 HYPERPROGRESSION IS NOT RESTRICTED TO ICB THERAPY

Although hyperprogression occurs in the settings of ICB treatment (PD1/PD‐L1 inhibitor treatment in particular) in most cases, patients receiving other therapeutic modalities showed similar tumor flares, including chemotherapy, radiotherapy, surgery, and targeted therapy. For example, one retrospective study indicated that HPD occurs in NSCLC patients receiving single‐agent chemotherapy, although its incidence was only one‐third of that observed in the cohort treated with anti‐PD‐1/PD‐L1 agents.^57^ Furthermore, chemotherapy might accelerate tumor cell proliferation in oropharyngeal cancer.^150^ For radiotherapy, the repopulation of surviving head‐and‐neck cancer cells can be induced by radiotherapy after the first 2 weeks of treatment.^151^ Similarly, ionizing radiation was reported to reprogram differentiated breast cancer cells into induced breast cancer stem cells, which showed enhanced tumorigenicity.^152^ Moreover, it has also long been debated whether surgical cancer resection could negatively affect tumor growth and metastasis. This is closely related to tumor dormancy modulated by resection, as verified by clinical and experimental conditions mainly in disease models of lung and breast cancers.^153^ Last but not least, the acceleration of tumor growth can also be found in patients receiving traditional immunotherapy, including the adjuvant IFN‐α^154^ and anti‐CD20 antibody,^155^, ^156^ or targeted therapy, such as RAF inhibitors^157^ and BRAF inhibitors,^158^, ^159^ or in patients with discontinuation of ALK‐tyrosine kinase inhibitors,^160^ EGFR‐tyrosine kinase inhibitors,^161^ and VEGFR‐tyrosine kinase inhibitors.^162^ To our knowledge, it is still unclear whether the underlying mechanisms of hyperprogression are the same or different in the context of different treatment choices. A thorough understanding of this phenomenon could help us to better manage this newly discovered detrimental response.

## 9 DIFFERENTIATION OF HYPERPROGRESSION AND PSEUDOPROGRESSION

However, when evaluating the efficacy of ICIs, it becomes challenging for clinicians to accurately distinguish hyperprogression from other harmless tumor responses, which are called pseudoprogression. Pseudoprogression refers to an initial increase in the size of tumor lesions with subsequent tumor shrinkage.^10^, ^67^ In contrast to hyperprogression, the initial growth of the tumor was proven to be attributable to necrosis and inflammatory cell infiltration by tumor biopsy.^163^ This phenomenon was first reported in ∼10% of patients with advanced melanoma during anti‐CTLA‐4 therapy.^10^ However, later, pseudoprogression was reported in multiple tumor types, such as melanoma,^164^ NSCLC,^165^ HNSCC,^166^ renal cell carcinoma,^167^ urothelial carcinoma,^168^ mesothelioma,^169^ and Merkel cell carcinoma.^170^ However, the rates of pseudoprogression never exceeded 20%.

Owing to the completely adverse underlying characteristics of hyperprogression and pseudoprogression, it is necessary to tell them apart to avoid prolonging ineffective treatment or premature cessation of efficacious treatment. However, utilizing pure medical imaging techniques according to the size‐based RECIST criteria for evaluation might lead to misclassification given that hyperprogression and pseudoprogression can both result in increased size in lesions at the early stage.^57^ Since we now know that pseudoprogression is characterized by infiltration of inflammatory cells instead of tumor cells in tumor sites compared with true progression, clinicians currently identify pseudoprogression mainly by lesion biopsy results before obtaining imaging information from patients. Fortunately, current studies have revealed many biological mechanisms of pseudoprogression, which have promising clinical application value.^171^, ^172^ Hyperprogression and pseudoprogression show differences in their progression timeline, biomarkers, histopathological features, and so on,^171^, ^172^ which can assist in their respective identification and are summarized in Table 4.

**TABLE 4:** Differences between hyperprogression and pseudoprogression

	Hyperprogression	Pseudoprogression
Characteristics	Accelerated progression outpaces the expected rate of tumor growth without shrinkage after treatment	Initial increased size or number of tumor lesions with subsequent tumor shrinkage after treatment
Predictors	Older ager, locoregional recurrence in the radiation field, high metastatic burden, MDM2 amplification, EGFR mutation, cfDNA copy number instability, and CD8^+^ T cell exhaustion	Decreased/low levels of ctDNA and IL‐8 levels, decreased genome instability number in ctDNA, decreased CXCL2, and increased MMP2
Histopathology	Primarily tumor cells present in enlarged tumor leisions	Necrosis and infiltration of inflammatory cells in leisions
Treatment option	Cessation of primary ineffective treatment	Continue primary efficacious treatment
Prognosis	Reduced survival durations	Favorable

Analysis of the quantitative alterations in ctDNA levels early in the course of the disease is considered a powerful adjuvant tool for standard imaging strategies to evaluate the responsiveness to ICI therapy.^173^ Lipston et al^174^ first pointed out that the levels of ctDNA in melanoma patients receiving ICI treatment were correlated with pseudoprogression. One patient with pseudoprogression showed undetectable ctDNA levels 3 weeks before clinical improvement, while three patients with progressive disease presented increased ctDNA levels. A case report of three patients with lung adenocarcinoma who developed pseudoprogression after anti‐PD‐1 therapy showed similar results in that when compared with the increase in patients with true progression, the level of KRAS‐mutated ctDNA was dramatically reduced to an undetectable level.^175^ Later, a study including 125 patients demonstrated that in melanoma patients who received PD‐1 antibody therapy, ctDNA profiles were capable of differentiating pseudoprogression and hyperprogression with high sensitivity (90%) and specificity (100%).^176^ All nine patients with confirmed pseudoprogression showed a favorable ctDNA profile, while only two patients with true progression showed such a profile.^176^ Longitudinal assessment of ctDNA could, therefore, be a powerful tool for determining tumor response, progression‐free survival, and overall survival, since enhancement of ctDNA expression might indicate a poor prognosis.^8^ Moreover, pseudoprogression manifested as a decrease in genome instability in ctDNA, unlike hyperprogression.^177^, ^178^ However, some patients with a tumor response may not show any reliably identifiable mutations when monitoring ctDNA levels, as reported by Gray et al.^179^ Given the availability of liquid biopsy samples in comparison to that of tumor tissue, more investigations are urgently needed to establish the specific correlations between the ctDNA level and tumor progression after ICI treatment.

IL‐8, a chemokine that can promote cancer progression by regulating the tumor immune microenvironment, has been verified to be closely related to tumor burden and treatment response in multiple cancer categories.^180^, ^181^ In melanoma and NSCLC, an early decrease in serum IL‐8 levels after PD‐1 blockade was associated with favorable prognosis and prolonged overall survival. Among 29 melanoma patients and 19 NSCLC patients treated with nivolumab or pembrolizumab, the serum concentration of IL‐8 showed a significant reduction in patients with the best response and a dramatic elevation in patients with true progression and nonresponders compared to the baseline level. The decrease in IL‐8 levels indicated prolonged overall survival and correctly reflected the true response of three patients with pseudoprogression.^182^ Notwithstanding, increased IL‐8 levels are reported to be irrelevant to progression‐free survival in NSCLC patients receiving anti‐PD‐1 therapies.^183^ Thus, detecting alterations in serum IL‐8 levels can assist in distinguishing true responses in patients from pseudoprogression.

ICIs function by modulating the immune responses of the human body, making the measurement of immune components a possible strategy to evaluate treatment efficacy. Matsuo et al^183^ found that among over 80 soluble immune mediators tested, continuous decreases in the levels of CXCL2 and increases in MMP2 were significantly correlated with progression‐free survival and were observed in all three NSCLC patients with pseudoprogression after anti‐PD‐1 treatment. CXCL2, along with its receptor CXCR2, promotes the progression of tumors by recruiting MDSCs to the tumor microenvironment.^200^ However, the precise mechanism underlying the role of MMP2 in the improvement of prognosis remains unclear.

Above all, to fully assess both the efficacy and safety of immunotherapies, all possible biomarkers should be considered in future studies to identify pseudoprogression from hyperprogression.

## 10 SUBSEQUENT MANAGEMENT OF PATIENTS WITH HPD

Patients with clinical indications of HPD should be reassessed early in the case of rapid progression. When patients are diagnosed, the first and most important thing is to inform the patients about this newly discovered paradoxical pattern of progression and the scientific uncertainty of the knowledge of this pattern to gain their consent and cooperation.^184^ Furthermore, inefficient primary treatments should be withdrawn immediately and replaced with other potentially more effective treatments in patients who are still in good clinical condition.^185^ Finally, according to the hypothesized underlying mechanisms, different strategies can be used for treatment of HPD. For instance, cytotoxic and antiangiogenic agents might be associated with improved efficacy when disease progression accelerates.^186^, ^187^ In the future, additional strategies can hopefully be discovered to overcome abnormal progression as our understanding of its pathophysiological mechanisms becomes increasingly clear. Nevertheless, clinicians should perform both radiological examinations and sample biopsies to help raise awareness of the underlying mechanism and better inform the clinical management of HPD.

## 11 CONCLUSIONS AND FUTURE DIRECTIONS

In the past few decades, even though ICI‐based therapies have radically transformed cancer treatment to improve the outcomes of patients owing to reduced toxicity, the available evidence now demonstrates the two opposing effects on tumor progression in a substantial proportion of patients treated with ICB therapy. However, there is no clear definition of hyperprogression or uniform method of assessing tumor growth, such as the determination of TGR, TGK, and TTF, which have not been extensively accepted by academia. Furthermore, whether PD‐1/PD‐L1 inhibitors could induce hyperprogression remains unknown. Perhaps tumor flare could also occur in some patients receiving other therapies or no therapy. Since the current studies on hyperprogression are retrospective studies with limited samples, more centers need to collaborate to obtain additional imaging scans and conduct prospective studies to truly evaluate this question. Despite the encouraging results obtained from several studies, no predictive biomarker of hyperprogression has been identified to date due to the discrepancies between different studies. As ICIs become increasingly prevalent in clinical practice, further studies are required to identify reliable predictors of HPD to screen patients with a high risk of developing this serious and life‐threatening immune‐related adverse event before the initiation of anticancer therapy. For such patients, ICI treatment should be administered with great caution or even avoided. Moreover, patients at risk of HPD should no longer be given ICB therapy. Instead, other potentially efficient treatments should be considered, such as salvage chemotherapy, to ensure the maximization of the possibility for patients to benefit from therapy after HPD. In addition, since the potential mechanisms of hyperprogression remain uncertain, comprehensive knowledge of immunological changes and mechanisms of HPD is urgently needed to resolve hyperprogression. Hence, more efforts are needed in the future to instruct clinical decision making for patients under ICB treatment.

## COMPETING INTERESTS

The authors declare that they have no competing interests.

## AUTHOR CONTRIBUTIONS

XJH and AQAL were major writers of the manuscript and designed the figures and tables. YNX researched appropriate references and edited the manuscript. XWW and YQW developed the structure of the article, reviewed, and edited the manuscript. All authors have reviewed and approved the manuscript prior to submission.
